# Using an intersectionality lens to explore barriers and enablers to hepatitis C point-of-care testing: a qualitative study among people who inject drugs and service providers

**DOI:** 10.1186/s12939-024-02209-0

**Published:** 2024-06-17

**Authors:** Guillaume Fontaine, Justin Presseau, Julie Bruneau, Cole Etherington, Isabella M. Thomas, Jui-Hsia Cleo Hung, Zack van Allen, Andrea M. Patey, Ayesha Kareem, Sameh Mortazhejri, Stine Bordier Høj, Geneviève Boyer-Legault, Jeremy M. Grimshaw

**Affiliations:** 1 Centre for Implementation Research, Clinical Epidemiology Program, Ottawa Hospital Research Institute, 501 Smyth Road, Ottawa, ON K1H 8L6 Canada; 2 Ingram School of Nursing, Faculty of Medicine and Health Sciences, McGill University, 680 Rue Sherbrooke O #1800, Montréal, QC H3A 2M7 Canada; 3 Centre for Clinical Epidemiology, Lady Davis Institute for Medical Research, Sir Mortimer B. Davis Jewish General Hospital, CIUSSS West-Central Montreal, 3755 Chem. de La Côte-Sainte-Catherine, Montréal, QC H3T 1E2 Canada; 4 Viral Hepatitis Clinical Research Program, Kirby Institute, UNSW Sydney, UNSW, Wallace Wurth Building (C27), Cnr High St & Botany St, Kensington, NSW 2052 Australia; 5 School of Epidemiology and Public Health, University of Ottawa, 600 Peter Morand Crescent, Ottawa, ON K1G 5Z3 Canada; 6 School of Psychology, University of Ottawa, 136 Jean-Jacques Lussier, Vanier Hall, Ottawa, ON K1N 6N5 Canada; 7 Research Centre, Université de Montréal Hospital Centre, 900 Saint Denis St, Montreal, QC H2X 0A9 Canada; 8 Department of Family and Emergency Medicine, Université de Montréal, 2900, Boulevard Édouard-Montpetit, Montréal, QC H3T 1J4 Canada; 9 School of Rehabilitation Therapy, Queen’s University, Louise D Acton Building, 31 George St, Kingston, ON K7L 3N6 Canada; 10 Direction of Community Services, CACTUS Montréal, 1300 Rue Sanguinet, Montréal, QC H2X 3E7 Canada; 11 Department of Medicine, University of Ottawa, 45 Smyth Road, Ottawa, ON K1H8M5 Canada

**Keywords:** People who inject drugs, Intersectionality, Harm reduction, Needle and syringe program, Hepatitis C, Implementation science, Point-of-care testing, Qualitative research

## Abstract

**Background:**

Hepatitis C virus (HCV) infection is a significant global health burden, particularly among people who inject drugs. Rapid point-of-care HCV testing has emerged as a promising approach to improve HCV detection and linkage to care in harm reduction organizations such as needle and syringe programs. The objective of this study was to use an intersectionality lens to explore the barriers and enablers to point-of-care HCV testing in a needle and syringe program.

**Methods:**

A qualitative study was conducted using semi-structured interviews with clients (people who inject drugs) and service providers in a large community organization focused on the prevention of sexually transmitted and blood borne infections and harm reduction in Montreal, Canada. An intersectionality lens was used alongside the Theoretical Domains Framework to guide the formulation of research questions as well as data collection, analysis, and interpretation.

**Results:**

We interviewed 27 participants (15 clients, 12 providers). For clients, four themes emerged: (1) understanding and perceptions of HCV testing, (2) the role of an accessible and inclusive environment, (3) the interplay of emotions and motivations in decision-making, and (4) the impact of intersectional stigma related to HCV, behaviors, and identities. For providers, five themes emerged: (1) knowledge, skills, and confidence for HCV testing, (2) professional roles and their intersection with identity and lived experience, (3) resources and integration of services, (4) social and emotional factors, and (5) behavioral regulation and incentives for HCV testing. Intersectional stigma amplified access, emotional and informational barriers to HCV care for clients. In contrast, identity and lived experience acted as powerful enablers for providers in the provision of HCV care.

**Conclusion:**

The application of an intersectionality lens provides a nuanced understanding of multilevel barriers and enablers to point-of-care HCV testing. Findings underscore the need for tailored strategies that address stigma, improve provider roles and communication, and foster an inclusive environment for equitable HCV care. Using an intersectionality lens in implementation research can offer valuable insights, guiding the design of equity-focused implementation strategies.

## Contributions to the literature

- Applies an intersectionality lens to identify barriers and enablers to hepatitis C point-of-care testing among people who inject drugs and service providers.
- Illuminates the impact of intersecting identities and structures of power on the experiences of individuals accessing hepatitis C testing and care.
- Highlights the importance of tailored implementation strategies to address stigma, enhance communication skills, and foster inclusive environments for equitable hepatitis C care access.
- Contributes to the evidence base for employing an intersectionality lens in implementation science to promote more equitable health services.
- Advances the global health agenda towards the elimination of hepatitis C as a public health threat.

## Background

Hepatitis C virus (HCV) infection poses a significant global health burden, particularly among people who inject drugs [1]. Globally, it is estimated that 14·8 million people inject drugs [2]. In high-income countries, up to 85% of new HCV infections occur in this population due to the shared use of contaminated needles and drug paraphernalia [1, 3]. The consequences of HCV infection include the development of liver disease, cirrhosis, cancer, and the potential need for transplantation [4–6]. Chronic HCV infection is also associated with significant physical and psychological burdens on affected individuals [7]. Physical health consequences can include chronic fatigue and increased susceptibility to opportunistic infections, impacting quality of life [8]. Psychologically, the stigma associated with HCV can lead to feelings of isolation and depression, further affecting mental health [9]. These burdens can strain relationships, hinder employment opportunities due to frequent medical appointments and reduced productivity, and ultimately diminish overall wellbeing [10].

Although no effective vaccine currently exists, the advent of highly-effective direct-acting antivirals with cure rates > 95% provide an opportunity to eliminate HCV globally [11–13]. In light of these therapeutic advances, the World Health Organization (WHO) has called for the elimination of HCV infection as a public health threat by 2030 [14, 15]. A major barrier to increasing uptake of HCV testing and treatment is that the current diagnostic pathway requires multiple visits (an HCV antibody test to confirm exposure, an HCV ribonucleic acid (RNA) test to confirm current infection, and one or more assessments to start treatment) leading to loss to follow-up and delays in treatment, which is exacerbated in higher risk groups [16]. Higher risk groups often face additional challenges such as unstable housing, substance use, and limited access to healthcare, making it difficult to comply with multiple visits [14]. Increasing access to testing and simplifying the diagnostic pathway will be critical steps towards achieving the WHO’s goal of eliminating HCV on a global scale [14].

The introduction of finger-stick tests capable of detecting HCV antibodies (results: 5–20 min) and HCV RNA (results: 60 min) at the point of care has revolutionized the clinical approach to HCV [17–23]. Point-of-care tests can be used by community workers and nurses, enable diagnosis and treatment in a single visit, increase testing acceptability, and reduce loss to follow-up [19–23]. Research has demonstrated reduced time to treatment initiation and high treatment uptake compared to standard diagnostic approaches [17, 18, 23–26], informing recommendations in the 2022 WHO guidelines on simplified service delivery and diagnostics for HCV infection [27]. The impact of point-of-care testing will be greatest in high HCV prevalence settings, such as needle and syringe programs who provide access to sterile injecting equipment [28, 29]. Harm reduction programs are critical intervention points because they provide services to people who inject drugs, a group that experiences higher rates of HCV due to practices such as needle sharing [28, 29]. However, there are numerous implementation challenges, including the revision of professional roles, training, quality assurance and integration of point-of-care tests into laboratory and public health surveillance frameworks [30–32]. A systematic, implementation science approach [33, 34] is essential to comprehensively identify factors that will limit or enable point-of-care HCV testing in community-based harm reduction organizations such as needle and syringe programs.

The traditional application of implementation science frameworks may overlook unique challenges faced by equity-denied groups, such as people who inject drugs, and the dynamics within settings serving these individuals [35–38]. People who inject drugs face stigma and discrimination which shape their interactions with healthcare systems, and access to care [2, 39, 40]. Recent shifts towards equity-focused implementation science underscore the importance of acknowledging and addressing these unique challenges to enhance health outcomes for all [35, 41–43]. The use of an equity-focused theoretical lens can complement determinant frameworks such as the Theoretical Domains Framework (TDF) by providing a deeper understanding of how intersecting social identities and structural inequities influence individuals' behaviors and attitudes, enriching the analysis of barriers and enablers to behavior change in healthcare settings [38, 42, 44, 45]. By considering the complex interplay of intersecting identities and structures of power within society (e.g., racism, classism, drug policy, stigmatization of HCV infection), an intersectionality lens has the potential to provide a more nuanced perspective on the factors influencing the implementation of point-of-care HCV testing within needle and syringe programs [46–48].

### Objective and research questions

This study aimed to use an intersectionality lens to explore the theory-informed barriers and enablers to point-of-care HCV antibody and RNA testing in a needle and syringe program in Montreal, Canada. We sought to answer the following research questions:

1. What are the barriers and enablers to point-of-care HCV testing in needle and syringe programs from the perspective of people who inject drugs and service providers?
2. How does social identity and structures of power related to the healthcare system and society intersect with and influence these barriers and enablers?

## Methods

### Study design and guiding framework

**Fig. 1:**
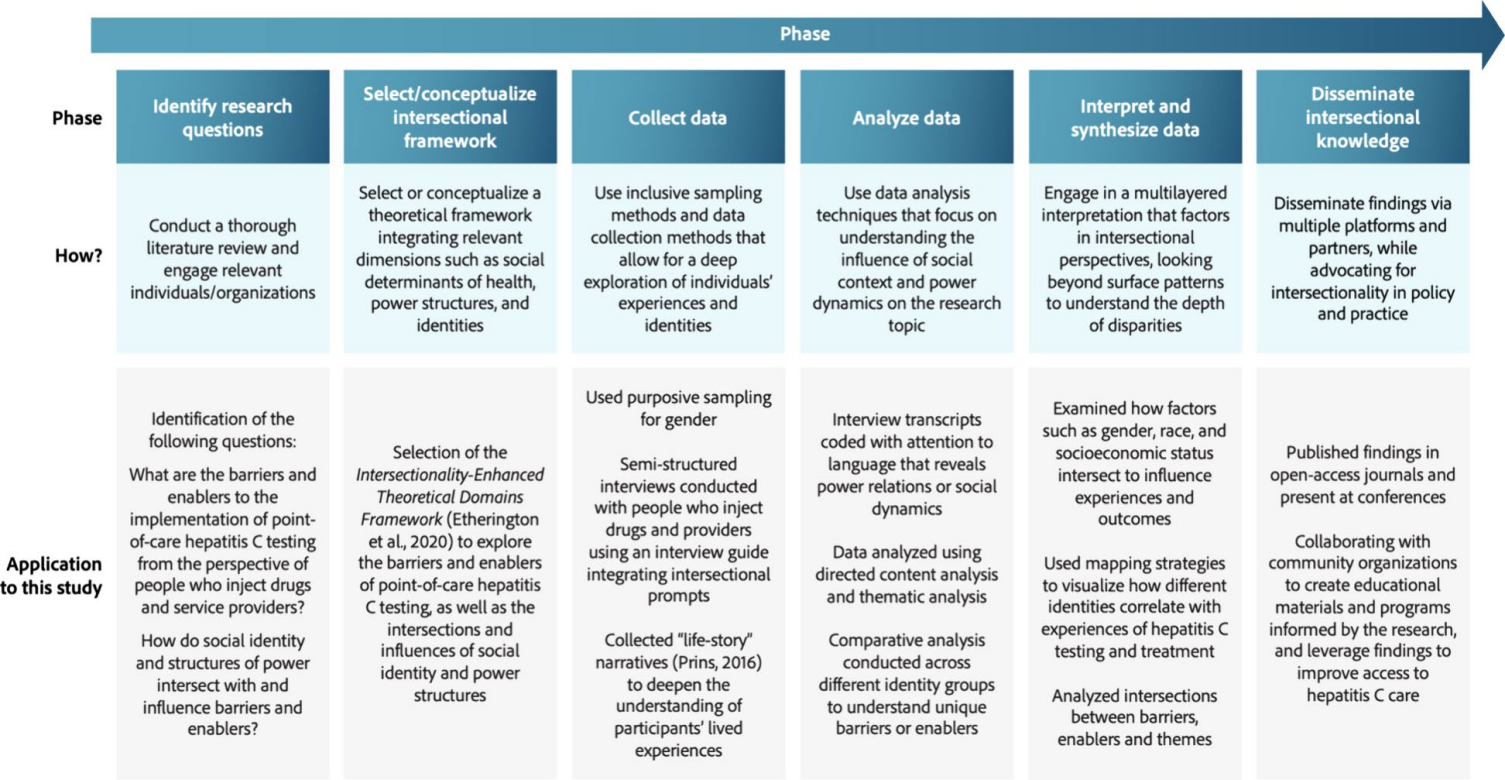
Using an intersectionality lens in implementation research

A theory-informed qualitative study was conducted using semi-structured interviews. As presented in Fig. 1, we adapted methodological guidance from different sources [45, 49–52] to apply an intersectionality lens across the different phases of the study. Intersectionality is an analytical framework rooted in critical social theory developed by black feminist and critical race scholars in the 1980s [53]. It recognizes that individuals possess multiple, intersecting identities that are influenced by power structures and social hierarchies [54]. Our approach aimed to determine how barriers and enablers to implementation of point-of-care testing were shaped by social identity factors in people who inject drugs and service providers, as well as structures of power (e.g., racism, classism, transphobia) related to the healthcare system and society. The Research Ethics Boards of the Centre Hospitalier de l’Université de Montréal (#21.197) and the Ottawa Health Science Network (#20,210,655-01H) approved the study.

We first identified research questions that were sensitive to intersectional perspectives. Next, we selected the intersectionality-enhanced TDF to guide data collection, including the development of an interview guide [55]. The TDF identifies 14 theoretical domains that influence behavior: (1) *Knowledge*; (2) *Skills*; (3) *Social/professional role and identity*; (4) *Beliefs about capabilities*; (5) *Optimism*; (6) *Beliefs about consequences*; (7) *Reinforcement*; (8) *Intentions*; (9) *Goals*; (10) *Memory, attention and decision processes*; (11) *Environmental context and resources*; (12) *Social influences*; (13) *Emotions*; and (14) *Behavioral regulation* [55]. Each theoretical domain may be an influencing factor (i.e., barrier or enabler) of the implementation of point-of-care HCV testing. These factors can then be targeted by implementation strategies and behavior change techniques suited to the influencing factor [56, 57]. The intersectionality-enhanced TDF [45] includes additional prompts that can be used to explore how social identity and structures of power intersect with the 14 domains. Our interview guide used some of these intersectionality prompts, which were modified to make them more naturalistic during the interviews. During interviews, in addition to TDF and intersectionality questions, we collected life-story narratives to better understand how individuals construct their identities based on various social categories [50]. Data analysis and interpretation were conducted using an intersectionality lens, recognizing the interconnected nature of various social structures and identities. Identity was seen as a narrative through which individuals both play the lead role and write the script, drawing upon different categories such as gender, class, and ethnicity.

### Setting and context

The study was conducted at CACTUS Montréal, a large urban community organization focused on harm reduction and prevention of sexually transmitted and blood-borne infections (STBBIs) among people who use drugs, sex workers, and transgender communities (https://cactusmontreal.org/en/). Established in 1989, it pioneered North America’s first needle and syringe program and employed more than 80 individuals at the time of the study. It is co-located with the largest supervised consumption site in the province of Quebec. Over the course of 2020–21, 70,939 individuals visited the organization through different programs such as the needle and syringe programs at two different sites, the youth program, and the social involvement program.

### Sample and recruitment

The sample for this study was composed of two groups: clients (people who inject drugs) and service providers. For clients, adult French- or English-speaking individuals with a recent visit (< 3 months) to the needle and syringe program and with a recent history (< 3 months) of injection drug use were eligible to participate. Individuals reporting a cognitive impairment or a health condition that might impede participation in this study were not eligible. For service providers, adult French- or English-speaking individuals working at CACTUS Montréal as peer workers, prevention officers, street workers or program coordinators. We used a modified version of the 10 + 3 decision rule to achieve an adequate sample for content validity, whereby we conducted 10 initial interviews followed by an additional two to three interviews until no new themes were identified [58]. We anticipated that 10 to 15 interviews per group (20 to 30 interviews total) would be sufficient. We used purposive sampling, aiming to include at least 30% of individuals identifying as women.

Identification of potential participants was conducted by CACTUS program coordinators and other staff members. A researcher (GF) was present onsite one to two days per week to describe the study, share posters containing a plain language explanation of the study and confirm the eligibility of individuals identified by CACTUS coordinators and staff. After confirming eligibility, we provided the informed consent form and obtained their signed consent.

To accommodate participant preferences, we scheduled individual in-person interviews at a suitable time in a dedicated private meeting room at the needle and syringe program, or via Zoom. The interviews were conducted by GF, a bilingual white male nurse who identifies as a member of the LGBTQ + community. At the time of the study, he was a postdoctoral researcher with expertise in qualitative research and was affiliated with the Ottawa Hospital Research Institute, but had no prior connections to the study setting. For people who inject drugs, this interview was the second of a series of two interviews focusing on HCV infection [59]. The first interview explored perceptions of HCV infection, while the second one explored barriers and enablers to testing. For service providers, no relationship had been established prior to the interviews. Each client and service provider received a compensation of $30 CAD upon the completion of the interview.

### Data analysis

A comprehensive coding manual was developed, incorporating standard intersectionality-enhanced TDF definitions, related theoretical constructs and decision rules to ensure consistency in coding. Each interview transcript was independently double coded using directed content analysis to categorize statements into domains of the intersectionality-enhanced TDF in NVivo (https://lumivero.com/products/nvivo/, version 12) by two researchers (GF, CE, IT, AK, ZvA, or CJHH). Any discrepancies were resolved through a consensus-building process. Representative themes were inductively developed to contextualize barriers and enablers to point-of-care HCV testing. The significance of key domains was assessed based on the strength of participants' beliefs within each domain, as well as their frequency and consistency across the data. The frequency metric indicated the number of participants contributing to a specific belief statement within each intersectionality-enhanced TDF domain. The data were analyzed with careful attention to language that may reveal social identity factors, power relations or social dynamics. Overarching themes were developed using thematic analysis, and the intersections between barriers, enablers, and themes were also analyzed. We used OpenAI’s ChatGPT 4 (https://chat.openai.com/chat) to enhance the coherence and readability of specific sections of the manuscript, as well as for proofreading purposes.

## Results

### Sample characteristics

Between October 29, 2021, and March 10, 2022, a total of 27 individuals were interviewed, including 15 clients and 12 providers. The characteristics of participants are presented in Table 1. Most clients were 40 years or older, 10 self-identified as men, and 13 self-identified as white. Stable housing was reported in only three participants. Self-reported drugs injected in the past three months included opioids (9/15), stimulants (3/15), and both opioids and stimulants (3/15). Twelve participants self-reported one or more past HCV infection(s). Of those, seven were treated with direct-acting antivirals, four were spontaneously cleared, and three were treated with interferon-based therapies. Most service providers were aged between 18 and 39 years old. Seven self-identified as female, and all self-identified as white. They held various positions, with half being prevention officers, and the rest distributed as program coordinators and street workers. Providers had a median work experience of 61.5 months at the current organization. All interviews were conducted in French. Interviews with clients had a median duration of 33.25 min, whereas interviews with providers had a median duration of 71.5 min. While the interview guide was of a similar length for both clients and providers, providers offered more detailed and extensive responses, resulting in longer interview durations. This discrepancy could also be attributable to the fact that some clients collected needles and syringes and were keen to leave quickly to use them. Providers did not have protected time to participate in the interviews and participated when possible during their day.

**Table 1:** Sociodemographic characteristics of participants (*N* = 27)

	**Clients (*n* = 15)**	**Service providers (*n* = 12)**
	**No. (%)**	**No. (%)**
***Age, y***		
18–39	5 (33)	7 (58)
> 40	10 (67)	5 (42)
***Gender***		
Women	5 (33)	7 (58)
Men	10 (67)	5 (42)
***Ethnicity***		
White	13 (87)	12 (100)
Indigenous	2 (13)	0
***Housing situation***		
No housing (i.e., street, park, bus station, car)	7 (47)	—
Transitional (i.e., hotel, shelter, rehabilitation center, couch surfing)	5 (33)	—
Stable (i.e., own apartment/house, parent’s house)	3 (20)	—
***Drugs injected, past 3 months***		
Opioid	9 (60)	—
Stimulant	3 (20)	—
Opioid & Stimulant	3 (20)	—
***Opioid agonist therapy, past 3 months***		
No	11 (73)	—
Yes	4 (27)	—
***Hepatitis C virus infection(s)***		
None	4 (27)	—
1	8 (53)	—
> 1	3 (20)	—
***Treatment(s) completed***		
None	7 (47)	—
Interferon-based (IFN) therapy	2 (13)	—
Direct-acting antivirals (DAAs)	5 (33)	—
IFN & DAAs	1 (7)	—
***Work title at the organization***		
Prevention officer	—	6 (50)
Program coordinator	—	4 (34)
Street worker	—	2 (17)
***Work experience at the organization***		
Experience in current organization, median number of months	—	61.5

### TDF domains and overarching themes in clients

**Fig. 2:**
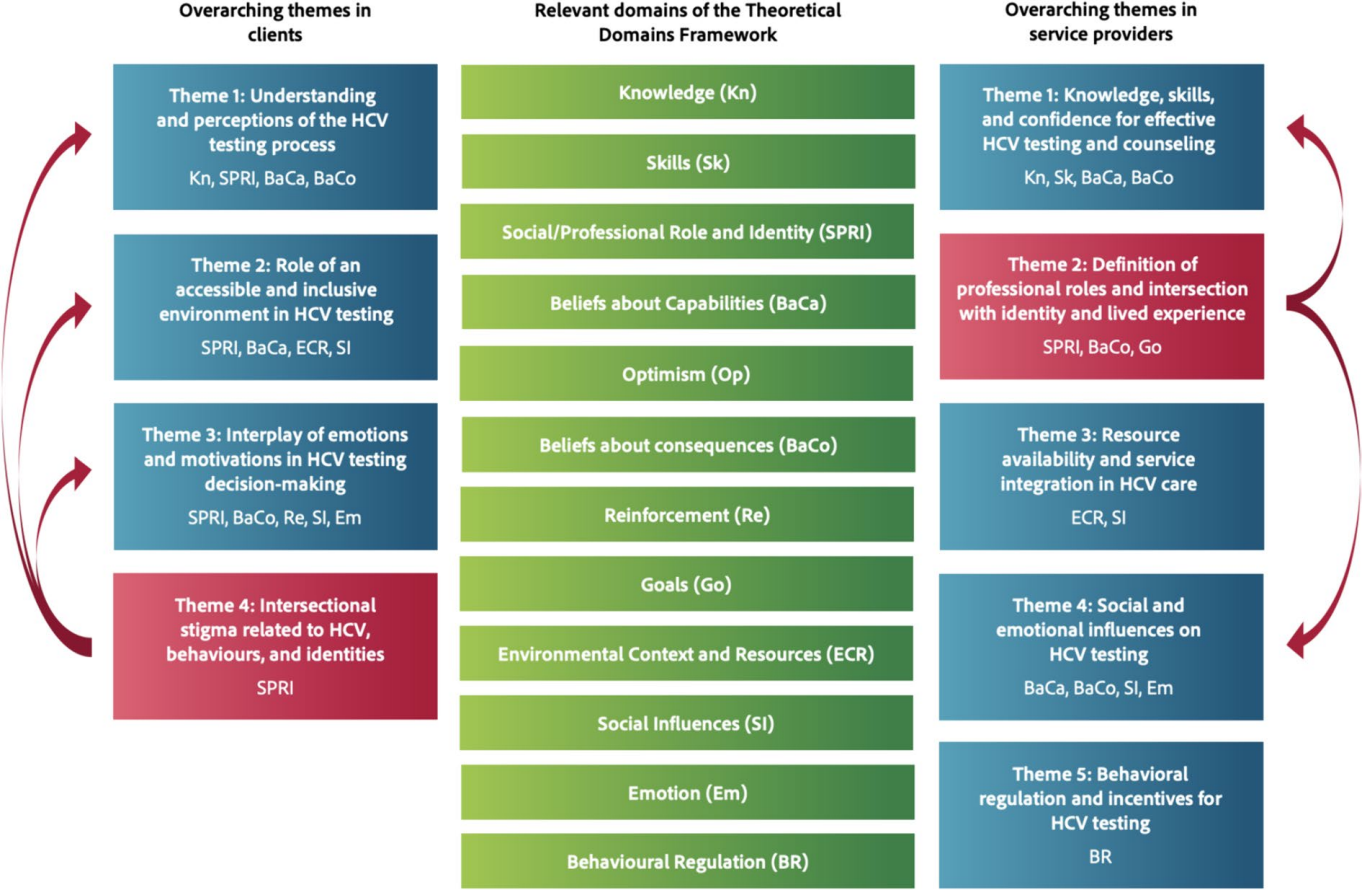
Overarching themes in clients and service providers influencing the implementation of hepatitis C virus (HCV) testing. The themes in red represent the primary themes emerging from the intersectional analysis, and the arrows represent their interrelations with other themes

In clients, our analysis identified that eight TDF domains (*Knowledge*; *Social/professional role and identity*; *Beliefs about capabilities*; *Beliefs about consequences*; *Reinforcement*; *Environmental context and resources*; *Social influences*; *Emotion*) were relevant for understanding barriers and enablers to implementing point-of-care HCV testing. These domains are highlighted in this paper in italics and parenthesis adjacent to relevant findings. Themes, subthemes, belief statements, sample quotes and key domains are presented in Table 2. Domain-specific barriers and enablers were synthesized into four overarching themes: (1) the understanding and perceptions of the HCV testing process, (2) the role of an accessible and inclusive environment in HCV testing, (3) the interplay of emotions and motivations in HCV testing decision-making, and (4) the impact of intersectional stigma related to drug use, identities, and HCV (see Fig. 2).

**Table 2:** TDF themes, belief statements and sample quotes linked to relevant TDF domains in clients

Belief statements	Frequency	Sample quote	TDF domain(s)
***Theme 1: Understanding and perceptions of the hepatitis C virus (HCV) testing process***			
*Subtheme 1.1: Knowledge about HCV testing*			
I have a good enough understanding of the procedure for HCV testing (*Enabler*)	12	“The last time I went to the screening center. My doctor had checked off everything, I told them that I wanted to know everything so, I go there, and they take eight vials approximately. […] We are in the waiting room, they come to get us, take the blood.” – HC13	Knowledge
I have some knowledge gaps about what exactly they test for, i.e., HCV antibodies, RNA (*Barrier*)	6	“What are we screening for? Is it really the virus? Or the anti-bodies that are taken from the test. A little more education. There’s a lot of information missing. I think that even the [service provider] didn’t know.” – HC16	Knowledge
*Subtheme 1.2: Influence of the HCV testing procedure*			
The traditional HCV testing procedure leads to long delays in getting your results (*Barrier*)	5	“We are not very informed on that. We do the test, and […] don’t really know what to expect.” – HC16	Beliefs about consequences
The traditional HCV testing procedure with venepuncture is complex (*Barrier)*	2	“I know it was complex, it took a long time. I don’t know if now it takes less time.” – HC02	Beliefs about capabilities
Point-of-care HCV tests are easy to do (*Enabler*)	3	“I think the test is easy to do, not complicated, just a few drops of blood and that’s it.” – HC01	Beliefs about capabilities
***Theme 2: Role of an accessible and inclusive environment in HCV testing***			
I would undergo HCV testing at the needle and syringe programme because I feel comfortable/at home (*Enabler*)	12	“Are there other things at Cactus that are make it convenient to get tested here?” “No, it’s more the environment. The only place that I see like home.” – HC27	Environmental context and resources; Beliefs about capabilities
The workers here are trustworthy and competent (*Enabler*)	8	“If I’m feeling comfortable, it is because the people here are trustworthy. I like the people here a lot. […] I would be super comfortable because I know they are discreet, and they have a big heart, and they really want to help. […] Well, there is a fundamental trust. It’s a place, it’s like my second family. So, I’m super comfortable and they spoke to me about it and since it came from people I trust, that I appreciate, well I take their advice. I’m confident that they don’t want to harm me.” – HC04	Social influences
It is easier getting screened at the needle and syringe programme than elsewhere because I come here for other reasons/activities of daily living (*Enabler*)	4	“I come here for other reasons as well, so that is more suitable, I come here for the materials, I come here because I have friends in the area, so I have more positive things… My day to day is closer to here.” – HC09	Beliefs about capabilities
***Theme 3: Interplay of emotions and motivations in HCV testing decision-making***			
*Subtheme 3.1: Emotional barriers to HCV testing*			
I am more anxious getting tested if I believe I have an active infection or high-risk behaviours (*Barrier*)	4	“Obviously when I was more at risk, like when I caught it, I was anxious for the results, yes, I was anxious." – HC09	Emotion
I was very anxious the first time I got tested (*Barrier*)	3	“The fact of being asked, I was incredibly stressed […] but then when it came to me getting the test, then I was extremely nervous because I had been informed about what hepatitis C was. […] I did not sleep for a week." – HC07	Emotion
If I accept testing, there will have to be a venipuncture at some point for RNA testing and that scares me (*Barrier*)	2	“Except that it annoys me a bit to do blood work. I can inject myself but to be pricked, it bothers me. It annoys me. It’s just that. You know, one shot you can take it but…as long as it’s one shot, you know. Often, they prick you in many places.” – HC13	Beliefs about consequences; Emotion
I would be worried about the impacts of HCV on my health if I knew I was positive (*Barrier*)	2	“I would certainly be worried for my liver, but I think it is to our advantage to know how healthy we are.” – HC05	Beliefs about consequences; Emotion
*Subtheme 3.2: Personal, social, and financial benefits of HCV testing*			
Receiving confirmation that I am not infected with HCV will provide me with peace of mind (*Enabler*)	9	“No, it is more the certainty of [knowing] if it is negative or positive because sometimes, we need to be reassured.” – HC04	Beliefs about consequences
I will be empowered to manage my health care and pursue treatment (*Enabler*)	8	“This gives me momentum. I really want to take charge of my life now. Once I’m there, things will change; I am working here. I am going to work here, I am going to be one of the staff later, I am telling you. What I mean is that I am going to get tested anyway. Even if I don’t use.” – HC19	Beliefs about consequences
Monetary incentives for getting tested and for getting results are very important (*Enabler*)	8	“I went more for the compensation they gave us than to have the results at that moment. […] I think that if I’d never been approached by someone who told me that we could be compensated to get a hepatitis C test, it would have taken a lot more time before I went.” – HC05	Reinforcement
It will prevent transmission of HCV to other people/loved ones (*Enabler*)	6	“I would want to know, so I don’t give it to others and to be able to help others. […] Yes, to protect others because if I have it, knowing that I have it, I would be careful not to give it to others” – HC07	Beliefs about consequences
I feel positive emotions when getting tested for HCV (*Enabler*)	4	“For me, I am happy when they send me the results – negative! It is not positive. So, I am happy then. A little thrill.” – HC19	Emotion; Reinforcement
***Theme 4: Intersectional stigma related to HCV, behaviours, and identities***			
Stigma related to HCV (*Barrier*)	6	“Well, other advantages [of testing], I came back normal. I became a normal person. I feel more normal than I was. My way of speaking is different. I’m less ashamed of myself. I have more social benefits. I know that I can be successful." – HC29	Beliefs about consequences; Social influences; Social/professional role and identity
I would be very comfortable getting tested here because there is no stigma, racism, or discrimination (*Enabler*)	3	“There’s none of that here. Here, you better not say you’re racist. Well, you can say it, but they will be on your case. So, it’s not a place for racists, or this or that. No, there’s no judgement on anything. They try to get results with people who go in circles.” – HC02	Social influences; Social/professional role and identity
The questionnaire about drug use alongside the traditional HCV testing procedure is stigmatizing (*Barrier*)	2	“There is a certain questionnaire about my private life […] It’s a questionnaire, they have a file that they read and in that file that’s where they find the questions that they ask, like where – what do I do during the week?” – HC07	Social/professional role and identity
I wouldn’t be comfortable getting tested here because of stigma related to my identity (*Barrier*)	2	“I don’t feel like [getting tested for HCV] here. Being judged. In fact, they don’t want to help me, they judge me, so no I don’t feel like it.” – HC18	Social influences; Social/professional role and identity

#### Theme 1: Understanding and perceptions of the HCV testing process

Most participants had a good foundational understanding of the HCV testing procedure, but knowledge gaps persisted among many about what exactly the HCV test screens for, such as antibodies or RNA (*Knowledge*). Many had concerns about elements of the traditional testing process, such as the need for venepuncture and the lengthy wait times for results, which can discourage individuals from pursuing testing (*Beliefs about capabilities, Beliefs about consequences*). Finger stick point-of-care HCV tests were perceived as enablers due to their simplicity and quickness (*Beliefs about capabilities*), suggesting a shift towards these testing methods could enhance the uptake of HCV testing.

#### Theme 2: Role of an accessible and inclusive environment in HCV testing

Most expressed that they ‘feel at home’ at the needle and syringe program, which would serve as a strong enabler, facilitating the decision to undergo HCV testing (*Environmental context and resources; Beliefs about capabilities*). The convenience of integrating testing with their routine activities and daily life would simplify the decision to get tested (*Beliefs about capabilities*). Moreover, the trustworthiness and competence of service providers have a positive impact, instilling confidence (*Social influences*).

#### Theme 3: Interplay of emotions and motivations in HCV testing decision-making

Emotional barriers to HCV testing were prominent, with increased anxiety cited as a common response when individuals perceive themselves at risk due to past behaviors (*Emotion*). This emotional response can also be present when individuals face the prospect of testing for the first time, marked by significant stress and concern over the unfamiliar process (*Emotion*). The necessity of venipuncture for RNA testing introduces an additional layer of fear and can act as a barrier due to the discomfort and invasiveness associated with blood draws (*Beliefs about consequences; Emotion*). Motivating factors were the assurance and peace of mind that come from a negative test result (*Beliefs about consequences*) and the ability to take proactive steps towards health and treatment if positive (*Beliefs about consequences*). Monetary incentives for testing and obtaining results were highly valued, acting as a practical reinforcement that can sway the decision to get tested (*Reinforcement*). Not all clients of the needle and syringe program received monetary incentives for HCV testing. Monetary incentives of variable amounts ($10–20$) were not provided routinely but were given in the context of ongoing research projects for getting Ab/RNA tested, and sometimes when the client came back to get the results of the test(s). Lastly, the motive of preventing transmission to others reinforced the personal and social benefits of getting tested, contributing to a positive emotional outlook (*Beliefs about consequences*; *Emotion*).

#### Theme 4: Intersectional stigma related to HCV, behaviours, and identities

In examining the narratives of clients concerning HCV testing with an intersectionality lens, a recurring theme across all domains is the profound impact of stigma. This stigma is multifaceted, or intersectional, manifesting not only in relation to the *health condition* itself (i.e., HCV) but also intersecting with stigma related to *behaviours* such as drug use, and *identities*, such as ethnicity, gender identity and socioeconomic status. An HCV + diagnosis is associated with the fear of stigma; however, many recognize that knowing their HCV status is a necessary step to access treatment, which can ultimately reduce self-stigma and lead to greater acceptance in their social circles (*Social influences*; *Social/professional role and identity*). Stigma associated with drug use has led to judgment and discrimination from healthcare providers and society, affecting trust in and access to healthcare services. Some reported that the testing and treatment procedure itself is stigmatizing, given that it often requires extensive information about one’s lifestyle and substance use.

An environment free from identity-based discrimination is perceived as crucial for point-of-care HCV testing, as it communicates an ethos of inclusivity and respect, encouraging individuals to engage with HCV testing services without fear of judgment (*Environmental context and resources; Social/professional role and identity*). Women, particularly those with non-normative appearances or from queer or trans identities, discussed having experienced gender-based discrimination, affecting their social status and access to healthcare. While many expressed that the needle and syringe program is an inclusive environment with trustworthy providers, some women do not feel welcome due to male-dominated gender dynamics at play, further limiting their access to essential health services and support. Accessibility and inclusivity were more frequently discussed by individuals who were more visibly marginalized compared to those who identified as cisgender, male, and white.

Across all participants, the upbringing and life background reveal some similarities that further illuminate the theme of intersectional stigma. Many participants have experienced significant life challenges, including homelessness, legal issues, and mental health struggles. These challenges often stemmed from or were exacerbated by their early life experiences, such as family dynamics or traumatic events. A common theme is the use of drugs as a form of escape or coping mechanism for dealing with personal difficulties or trauma. Participants often emphasized the importance of community organizations for social interaction, support, and access to health services. These organizations provide a sense of belonging and acceptance that is crucial for their well-being. Despite their struggles, many express a desire for autonomy and take proactive steps to manage their health, including getting tested for HCV. They also demonstrate a sense of responsibility for their health and the well-being of others.

As presented in Fig. 2 and the interrelation of the themes demonstrated by the arrows, this intersectional stigma can compound various barriers related to accessing HCV care. The understanding and perceptions of HCV testing (Theme 1) are influenced by the stigma associated with HCV and drug use, which can exacerbate knowledge gaps and fears about the testing process. Individuals might be hesitant to seek testing due to the stigma of being labeled as a drug user or having HCV, and the traditional testing process, which involves questioning about drug use and lifestyle, can feel invasive and judgmental, further deterring individuals from getting tested. The perception of the accessibility and inclusiveness of the testing environment (Theme 2) is also impacted, as stigma can significantly impact how comfortable and welcome individuals feel in environments. For example, as described previously, women may feel unwelcome in male-dominated settings, affecting their willingness to access testing services. The interplay of emotions and motivations in HCV testing decision-making (Theme 3) is also intertwined with stigma. Emotional barriers to HCV testing, such as anxiety and fear, can be heightened by anticipating stigma related to an HCV + status, deterring individuals from getting tested. Addressing intersectional stigma is therefore crucial for improving the HCV testing experience and ensuring that individuals from diverse backgrounds feel supported and empowered to access testing and care.

### TDF domains and overarching themes in service providers

In service providers, our analysis showed that 10 TDF domains (*Knowledge*; *Skills*; *Social/professional role and identity*; *Beliefs about capabilities*; *Beliefs about consequences*; *Goals*; *Environmental context and resources*; *Social influences*; *Emotion; Behavioural regulation*) were relevant for understanding and addressing barriers and enablers to implementing HCV point-of-care testing. Themes, subthemes, belief statements, sample quotes and key domains are presented in Table 3. Domain-specific barriers and enablers were synthesized into five overarching themes: (1) the knowledge, skills and confidence for effective HCV testing and counseling, (2) the definition of professional roles and their intersection with social identity and lived experience, (3) the availability of resources and integration of services in HCV care, (4) the social and emotional influences on HCV testing, and (5) behavioral regulation and incentives for HCV testing (see Fig. 2).

**Table 3:** TDF themes, belief statements and sample quotes linked to relevant TDF domains in service providers

Belief statements	Frequency	Sample quote	TDF domain(s)
***Theme 1: Knowledge, skills and confidence for effective HCV testing and counseling***			
*Subtheme 1.1: Knowledge about HCV testing*			
Offering HCV testing/treatment will improve the health and quality of life of individuals (*Enabler*)	8	“I’m going to [offer HCV testing] for sure because the earlier you get treated, the fewer the complications […] The faster it happens, the better it happens, the better the chance to re-affiliate people to society, to the healthcare system, and to be able to offer a treatment team and move on.” – HC14	Knowledge; Beliefs about consequences
I have the knowledge base to explain HCV tests, including the difference between Ab and RNA testing (*Enabler*)	7	“Well, one tests whether you have antibodies and the other one will test whether the virus is active in your body.” – HC21	Knowledge
Offering HCV testing/treatment will contribute to elimination by increasing testing, reducing transmission (*Enabler*)	6	“If we want to stop the epidemic of hepatitis C, are there many solutions? Hepatitis C isn’t going to be stopped by your prayer beads, it’s about screening, and awareness and treatment… Leveling things out.” – HC08	Knowledge; Beliefs about consequences
I don’t know much about HCV testing (e.g., difference between Ab and RNA testing) (*Barrier*)	5	“Especially in people who use drugs or are homeless, I don’t know if you are supposed to do that, the RNA test right after – I don’t understand exactly what the test entails, let’s say you need to do a second test, a second meeting.” – HC25	Knowledge
Offering HCV testing/treatment will lead to economic and societal benefits (*Enabler*)	4	“Obviously for society, the more people we treat, the cheaper it gets for society. I like when my taxes are used properly, so obviously for society, I try to mention that.” – HC14	Knowledge; Beliefs about consequences
I don’t know much about HCV rapid point-of-care tests (*Barrier*)	2	“I know that at one time there were rapid tests. At the end of a finger but is it efficient or not?” – HC30	Knowledge
*Subtheme 1.2: Essential skills for HCV testing and linkage to care*			
Lack of skills to effectively integrate the offer of HCV testing in workflows (*Barrier*)	8	“It would be nice to organize training under the angle of dialogue between the nurses and the workers, as we have very close day-to-day practices, […] I think, as I said, it would facilitate that discussion and those exchanges and experience-based knowledge, which are very important.” – HC10 “I think [we need specific training on] approaches based on ethnocultural relationships, trans people, intersex people, queer people, people with disabilities.” – HC25	Beliefs about capabilities; Skills
I feel comfortable in offering HCV testing (*Enabler*)	6	“I have the tools – I’m lucky, no, I worked for it, I educated myself […] it’s easy for me to de-dramatize and explain the process, I feel I have the tools to answer those questions. And yes, there is always something beyond my abilities, but I have a phone, I can call [name], I can go get the information, I’m not going to leave that person alone, so they are really going to get the information quickly and accessibly.” – HC14	Beliefs about capabilities; Skills
Problem-solving skills to provide tailored support throughout all stages of the testing process (*Enabler*)	5	“We’ll look, with them, at all the places where they offer [HCV testing], the resources, depending on what their connections are already with resources or the hospital system. Do they have follow-ups in certain places? So, we’ll look at what is more suitable for them. What is more practical in their situation.” – HC28	Skills
Effective pre- and post-test counseling skills are essential for engaging individuals in care and delivering HCV test results with empathy and support (*Enabler*)	5	“I think you have to be comfortable with the announcement of the results. […] Will you be able to support the person and direct them? Because you are opening a door but you need to close it properly because you can’t say it’s 5 PM and I’m done working and you are positive, bye! […] But I think we really need to support the person, also be able to support a person in crisis, because a crisis is not just screaming and crying, you can also have a silent crisis – they are the worse by the way – so knowing the psychological state of the person. I’ve often given people results, and the people were completely disorganized, and they were just like “I’m just going to throw myself in front of a car”.” – HC15	Skills
Empathy and relational skills are essential to develop a trusting relationship (*Enabler*)	4	“It’s always better to know the person on a minimum level to start [talking about HCV testing], which does involve a certain intimacy – we are close enough to their intimacy when it comes to injecting and fixed sites, so I think it’s important to have a connection […] I would say, undeniably, the relationship with the person.” – HC10	Skills
Motivational skills are essential to motivate individuals to get tested and follow through with treatment (*Enabler)*	4	“The biggest quality is accepting the person as they are and where they want to go. I have zero expectations and 100% hope. If I have no expectations toward someone then I am never disappointed and when you are never disappointed, the other isn’t in a place to have to perform, to make you happy, to do good, to get their stickers; they are who they are, with what they can be.” – HC08	Skills
Communication skills are essential to de-dramatize HCV and use positive framing of HCV testing (*Enabler*)	3	“I’ve said “I think you might have caught a little something that needs to be treated quickly so that you don’t give it to somebody else, apparently it’s not a gift”, […] We have to de-dramatize and accompany them because they are afraid […] You have to level it out, because otherwise hepatitis C is scary, what does it do? My liver is done for! I’m going to have cirrhosis! […] I offer it based on who has a girlfriend, who wants one, a boyfriend or whatever, a lover; can we get a tune-up before we go further?” – HC08	Skills
***Theme 2: Definition of professional roles and intersection with social identity and lived experience***			
*Subtheme 2.1: Role clarity and task sharing*			
Offering HCV testing and support individuals for testing is part of my role as a harm reduction worker (*Enabler*)	9	“It’s part of my job. In fact, as I was saying, since I’ve been here, my main objective has always been prevention and treatment of STBBIs. […] Testing is the reason we are here, basically. The rest is a lever or a method of prevention.” – HC23	Social/Professional role and identity
HCV is a priority, and my main objective is prevention and treatment of STBBIs (*Enabler*)	5	“My main objective has always been prevention and treatment of STBBIs.” – HC23	Goals
There is a need for task sharing between professionals and clarity in roles (*Barrier*)	5	“We cannot make appointments for HCV testing as a [harm reduction] worker, each time we have to transfer to the nurses.” – HC10	Social/Professional role and identity
HCV is one priority amongst many competing priorities (*Mixed*)	5	“I’m motivated when the person is motivated to hear about [HCV testing]. Often there’s an interest for that. But often there are other issues. There are other things that are a priority for them. They’re positive but sometimes, that’s it, it will be put off to when other things will be stabilized. When they are out of certain problems in their lives or certain periods more problematic.” – HC28	Goals
Empowering and developing the skills of workers in harm reduction (*Enabler*)	4	“That’s an advantage for me, but also a question of gratification, so for me, taking up a new practice or having the ability to take part in that screening process, is something very positive I think” – HC10	Beliefs about consequences
Offering HCV testing and support individuals for testing is not part of my job description (*Barrier*)	3	“I find it important to master hepatitis C but at the same time, I don’t think that it’s my role at [organization]. Besides directing people, it’s delicate as a subject because I don’t want, it’s delicate, the approach is delicate I don’t want to lecture. I don’t want…they get stoned to forget, eh? To not be in touch with…they abandon themselves. They destroy themselves. They don’t like themselves. So, to encourage them in that, it’s confronting for them. That’s my experience. It’s that, to stop playing savior. It’s to build and organize according to their needs, their requests.” – HC24	Social/Professional role and identity
HCV is more of a priority for some individuals (e.g., pregnant women, mental health issues, coinfections) (*Enabler*)	3	“All people with mental health conditions, mental disability problems, that is a priority for them because the ability to make a decision is difficult […] you need accompaniment to get people to go through the process.” – HC08	Goals
HCV is not my priority (*Barrier*)	2	“In theory, I’m very motivated. In practice, we are drowning in our daily tasks that unfortunately, it’s not my priority. My priority will be to maintain the service offering, its continuity and all that, you know.” – HC29	Goals
*Subtheme 2.2: Leveraging lived experience to intervene*			
I use my lived experience of drug use, homelessness, or HCV to intervene with individuals about HCV (*Enabler*)	8	“That’s the whole point, it’s to use my experience and my life to intervene with people. I use it as a work tool, ensuring that interventions are best adapted to people’s realities. […] Just the fact I understand, intimately, the daily life of someone in that situation and being able to share experiences or having a better contact with the street and being able to communicate the information. Of course, it has a great influence – as much for, I don’t know, an appointment, someone who uses opiates by injection, if you give them an appointment in two weeks at 8 AM, they will never be there. For example. Of course, it can be to help bridge communications between them and professionals or people they are trying to communicate with.” – HC21	Social/Professional role and identity
***Theme 3: Resource availability and service integration in HCV care***			
*Subtheme 3.1: Availability of staff and healthcare providers*			
There is a lack of staff and a lot of turnover (*Barrier*)	9	“There’s a big turnover of staff. It’s hard. […] I can’t either forget the budget, all the story of human resources, the difficulty of human resources. To keep, to retain staff. You know, it’s systemic. The general manager asked for grants to the government, to society focused on that theme, that social issue.” – HC24	Environmental context and resources
Lack of dedicated/permanent healthcare provider onsite (*Barrier*)	5	“We should have nurses who are available at all times, on-site […] you are ready now, it needs to be now! If it’s not now, unfortunately it will not happen. Again, the notion of time is very different, reality is so different when you live in the streets or in survival mode or using or whatever.” – HC21	Environmental context and resources
Nurses at the supervised injection site are available to offer HCV testing (*Enabler*)	3	“There are nurses at the Supervise Injection Site – if I know for a fact that the person uses drugs, I send them there, but if I don’t think the person uses drugs, then I’m not going to send them there, which has nothing to do with their world.” – HC25	Environmental context and resources
*Subtheme 3.2: Organizational processes and resources*			
Easy communication with and favourable influence of decision-makers (*Enabler*)	7	“I can speak directly to the Director, he has knowledge about drugs and pharmaceuticals, and when I have questions about that, whether it’s anything, I can make an appointment with my Director and we can chat about that and I will use what he knows.” – HC14	Environmental context and resources; Social influences
Lack of rapid point-of-care testing (*Barrier*)	7	“I think it’s important to offer [HCV testing] that is easy, quick and accessible because then it’s much easier: you have a positive result, okay, go, don’t panic, don’t freak out, we need a confirmation – but at least you can offer a fast answer” – HC14	Environmental context and resources
Lack of service stability (*Barrier*)	7	“[The major barrier] is the lack of service stability. […] that the main problem is going to occur when things are not done immediately. We have trouble scheduling appointments.” – HC23 “There is not always a nurse who is available. There is not always a clinical nurse who has the possibility to take blood. So, for several years now, I find that the offer has deteriorated at the supervised injection site. Therefore, we are more to reference and orientation elsewhere. Because we can’t seem to provide that service, that we should provide.” – HC29	Environmental context and resources
Lack of organizational processes/guidelines for HCV testing (*Barrier*)	6	“It’s something we talk about sometimes in team meetings but there is no protocol, no…it’s just case studies, by interventions that we did or situations that we experienced. But otherwise, no, there are no guidelines.” – HC28	Environmental context and resources
Lack of appropriate space to ensure confidentiality of HCV testing (*Barrier*)	5	“What would facilitate things is to have somewhere confidential to conduct the tests. […] We have one of the biggest teams and we are stuck in a Harry Potter closet – there is no room.” – HC25	Environmental context and resources
HCV testing often requires a Medicare card, which makes it really difficult for some people (*Barrier*)	5	“We have a nurse who comes to Cactus once a week for testing – she comes from a private clinic, but you need the Medicare card in that case, so depending on the situation…” – HC21	Environmental context and resources
***Theme 4: Social and emotional influences on HCV testing***			
Influence of the relationship with the individual is significant (*Mixed*)	7	“I think a lot of it is about the relationship of trust you have with the person, which will make a difference. […] It depends on the relationship you have with the person and their needs In some cases, there are kids who – I did not wait long enough to create that relationship of trust – so there was no result, it did not have a negative impact, but it just did not work because there was not that relationship of trust” – HC21	Social influences
Offering HCV testing might negatively impact my relationship with clients (*Barrier*)	7	“Could [offering HCV testing] have negative impacts?” “Maybe people will find me annoying, but I don’t think it would get to the point of breaking the relationship.” – HC21	Social influences; Beliefs about consequences
It is often difficult to engage with clients about HCV testing/we don’t have control over what happens to people (*Barrier*)	6	“Getting in touch with them [is difficult]. You do the first test, and then you don’t see them for six months. Today, one of them had a test, he was positive, and he went to prison for four months. We know he is in prison, but we are like…” – HC08	Beliefs about capability; Social influences
Favourable peer influence and support about HCV (*Enabler*)	6	“I helped my colleague for her “Hepatitis C Day,” I made flyers, I have some knowledge which meant that I often worked as a partner, or we talked about it because the people I was close to were in it. Obviously, this influenced me, but I also know that when you work as a team, you often have one person who is a master, a specialist, we all have our strengths and weaknesses” – HC14	Social influences
Emotional and psychological impacts on workers (*Barrier*)	6	“Giving a positive diagnostic information to someone about a virus which is relatively destructive, it’s not easy. It a psychological mental burden [for us] on top of that.” – HC29	Emotion; Beliefs about consequences
Behavioral, psychosocial and communication challenges (*Barrier*)	3	“Sometimes there’s something with people, you are not comfortable, we have more and more aggressive people, verbally, I’ve never been hit or anything, but sometimes they yell.” – HC15	Social influences
Success with HCV testing can bring us positive emotions (*Enabler*)	4	“Excited. Positive. Excited to be able to do something more, excited about helping my community, excited about learning something new. Positive because I think it’s going to be positive to be able to offer a service and at the same time empowering because I will be able to empower my community by helping them so they can be screened more comfortably.” – HC25	Emotion
***Theme 5: Behavioral regulation and incentives for HCV testing***			
Having a network of contacts for HCV testing is helpful (*Enabler*)	4	“It’s always the personalized referral, so if I tell them to go to this hospital, they won’t go. You have to go see [this person], at this hospital, third floor, and I call her and she’s waiting for you. […] The way we work here is we know who high risk users are, and we start talking “do you know [this worker]?”, Everybody knows [this worker]. “She is in a hepatitis C program, let me call her for you to see if she’s available, because it’s nice to get it done again”, and then she comes down as fast, and then we get started.” – HC08	Behavioural regulation
Financial compensation for individuals get screened, getting their results, being linked to care (*Enabler*)	4	“Depending on where we come from, paying people to offer them a service may seem a little bit disconnected, but on a greater scale, it is much cheaper to pay people 20 bucks to get tested, rather than them not knowing and giving it to other people, and then we have to pay for the treatment for all those people.” – HC21	Behavioural regulation
Bundling HCV testing with interventions addressing other needs (*Enabler*)	3	“If you tell me you are looking for a place to stay right now, I’m going to ask you if you had hepatitis C testing and I may have a potential place to offer. […]I will use hepatitis C for anything that will benefit me managing more than one thing. I would say it’s always part of a combination.” – HC14	Behavioural regulation
Rewards and/or objects to promote screening (e.g., lighter, hygiene kits, backpacks, coffee, pastries) (*Enabler*)	2	“I think this was for hepatitis C, there were lighters with logos and information on them. There were calling cards with information about screening and hepatitis C – we still have some, and we still print them. Generally, that is helpful, every time, even if the person hasn’t discussed it with me at all, but “it’s possible that you might talk to a woman who will tell you about hepatitis C, please know that she will give you all the information about screening and the phone number is on the card”. That’s an alternative way of discussing it. Tools like that work well.” – HC21	Behavioural regulation
Using opportunities/healthcare needs are a window of opportunity to offer screening (*Enabler*)	1	“Now I have a guy with a big abscess, so if he’s got an abscess this big, […] it means he injects in less than favourable conditions, so he is at high risk. […] That is my lever that is going to make me go, okay, you have an abscess, so it’s on his left hand so he can have myocarditis, and because you have an abscess, you may have got other things, so then we do the funnel, so I go through it, this is eliminated, we can eliminate hepatitis C, we are going to do a hepatitis C test, we are going to do a checkup, so we can check that off, so I go through it as if hepatitis C is not dangerous, as though it’s just part of STIs.” – HC08	Behavioural regulation

#### Theme 1: Knowledge, skills, and confidence for effective HCV testing and counseling

Most service providers believed that offering HCV testing and treatment would enhance the health and quality of life of individuals, and many highlighted broader benefits such as HCV elimination and economic benefits (*Knowledge*; *Beliefs about Consequences*). Providers recognized the importance of understanding antibody and RNA testing to accurately explain these tests to clients (*Knowledge*). The lack of skills to effectively provide HCV testing was identified as a major barrier (*Beliefs about capabilities*; *Skills*). Possessing good problem-solving skills, and effective pre- and post-test counseling skills were seen as enablers, contributing to the engagement of individuals in care and the empathetic delivery of test results. The importance of empathy, relational skills, motivational skills, and communication skills in developing trust and positively framing HCV testing was also emphasized (*Skills*). Consequently, many identified a need for practical training and training focused on ethnoculturally sensitive approaches and inclusive practices for effectively engaging transgender, intersex or queer individuals, and people with disabilities, in testing.

#### Theme 2: Definition of professional roles and intersection with social identity and lived experience

While some perceived that offering HCV testing is not a clear component of their job description, most agreed that the prevention and treatment of STBBIs, including HCV, was a priority and integral to their professional role (*Social/Professional Role and Identity*; *Goals*). However, most highlighted the need for redefinition of professional roles and task sharing to allow non-healthcare providers to play a bigger role in providing HCV testing. Importantly, most providers highlighted how crucial it is to leverage their social identity and lived experience in intervening with clients (*Social/Professional Role and Identity*). Providers often leveraged their own identities, for instance as members of the queer or transgender communities, and lived experiences, such as personal histories of drug use, homelessness, or HCV infection, to enhance communication and relate more authentically with clients, integrating their professional role with their social identity (*Social/Professional Role and Identity*).

#### Theme 3: Resource availability and service integration in HCV care

Many providers pointed out that, although nurses were available at a supervised injection site near the needle and syringe program to conduct HCV testing, the absence of dedicated or permanent healthcare providers directly on-site at the needle and syringe program was a significant barrier to testing (*Environmental context and resources*). Additionally, the shortage of staff and high turnover rates were identified as obstacles, leading to the need for continuous training and resulting in a loss of expertise in HCV care. Another major barrier was the absence of finger stick rapid antibody and RNA point-of-care HCV testing, which were described as crucial interventions to minimize loss to follow-up and ensure immediate linkage to care (*Environmental context and resources*). Many highlighted that without finger stick point-of-care tests, individuals may not receive timely results, increasing the likelihood of disengagement from care and reducing the chances of successful treatment and management of HCV. A key enabler was easy communication with and favorable influence of managers within the organization (*Environmental context and resources*; *Social influences*).

#### Theme 4: Social and emotional influences on HCV testing

Service providers emphasized the significant role of social and emotional factors in HCV testing. The trust and rapport built between providers and clients were identified as crucial enablers, fostering a conducive environment for discussing HCV testing (*Social influences*). However, concerns were raised about the potential negative impacts on these relationships if HCV testing is perceived as intrusive or judgmental (*Social influences*). Engaging clients in HCV testing discussions was highlighted as challenging, particularly due to the transient nature of some clients' lives, such as those experiencing homelessness or incarceration. Providers stressed the importance of peer support and positive influences within the client community to encourage testing uptake (*Beliefs about capability*; *Social influences*). The emotional and psychological impacts on providers themselves were also noted, particularly when delivering positive HCV diagnoses. Providers expressed a need for support and strategies to manage the emotional burden associated with this aspect of their work (*Emotion*).

#### Theme 5: Behavioral regulation and incentives for HCV testing

Service providers highlighted the importance of behavioral regulation strategies and incentives to promote HCV testing. Having a network of contacts for HCV testing and linkage to care was identified as an enabler, facilitating referrals and collaboration between different healthcare providers and services (*Behavioural regulation*). Financial incentives for clients to get screened, receive their results, and engage in care were seen as effective motivators. Providers noted that such incentives could increase testing uptake and ultimately be cost-effective for the healthcare system. Bundling HCV testing with other interventions addressing clients’ needs was recognized as a practical approach. Providers also mentioned using rewards or tangible items, such as hygiene kits or coffee vouchers, to promote screening. These small incentives were seen as helpful tools to initiate conversations about HCV testing and encourage participation (*Behavioural regulation*). Finally, providers emphasized the importance of seizing opportunities when clients present with healthcare needs, such as abscesses, to offer HCV testing. By linking testing to immediate health concerns, providers could frame HCV testing as a routine part of healthcare rather than an isolated or stigmatized procedure (*Behavioural regulation*).

As presented in Fig. 2, Theme 2 intersects with other themes, particularly Themes 1 and 4. The social identity and lived experience of service providers informs the knowledge and skills necessary for effective communication about HCV testing and counseling (Theme 1). Social identity and lived experience represent active components of their professional practice and enhance their skills and ability to deliver empathetic care and build trust. Their identity and lived experiences also provide them with unique insights into the social and emotional challenges faced by clients (Theme 4). This allows them to tailor their HCV testing interventions to account for the social and emotional dynamics at play. For instance, a provider who has experienced homelessness may better understand the challenges faced by a client without stable housing and can adjust their approach accordingly.

## Discussion

In this qualitative study, we applied an intersectionality lens alongside the TDF to investigate barriers and enablers to the implementation of point-of-care HCV testing in needle and syringe programs from the perspectives of people who inject drugs and service providers. The intersectional approach allowed for a nuanced understanding of how intersecting social identities and structures of power influence the implementation process. This understanding helped to identify how these factors compound key barriers or enhance enablers. The findings revealed four overarching themes for people who inject drugs and five for service providers. These themes not only illuminate the intricate dynamics at play but also point to a substantial window of opportunity for enhancing HCV testing and care, provided that tailored implementation strategies are employed to address the identified barriers and leverage the enablers.

Our findings indicate that people who inject drugs perceive the traditional testing process as a significant barrier to HCV testing and treatment uptake due to the need for venipuncture and lengthy wait times, aligning with existing literature [19, 60–63]. In contrast, the simplicity and rapid results of finger stick point-of-care antibody and RNA HCV tests are viewed as enablers [19, 60–63]. Also aligning with previous research in Australia is the perception of the needle and syringe program as an ideal environment to access HCV testing, with most individuals feeling at home, trusting service providers, and highlighting that it would align with their activities of daily living [16, 62, 64, 65]. Our study expands current knowledge on the intersectional stigma faced by people who inject drugs in traditional healthcare settings, correctional settings and society [59, 66–72], and how this stigma can compound key barriers related to accessing HCV care. The application of an intersectionality lens offers a nuanced understanding of how multiple facets of stigma related to HCV, behaviors, and identities intersect in the lives of people who inject drugs, leading to cycles of internalization and reinforcement by healthcare teams and the public. As a result, several barriers to HCV testing are exacerbated, including emotional barriers (e.g., heightened anxiety and fear linked to testing), access barriers (e.g., avoidance of traditional healthcare settings) and informational barriers (e.g., misconceptions and lack of knowledge about HCV, testing and treatment). Addressing intersectional stigma is therefore crucial for improving the HCV testing experience and ensuring that individuals from diverse backgrounds feel supported and empowered to access testing and care.

Providers expressed a lack of knowledge about HCV testing, particularly regarding rapid point-of-care antibody and RNA tests, highlighting the need for thorough theoretical and practical training. This is consistent with the general practice of providing extensive training to providers for administering point-of-care antibody and RNA HCV tests [18, 24, 73]. Additionally, there was a notable emphasis on the importance of training in pre- and post-test counseling to ensure comprehensive care. As in previous research [74], the need for task-shifting and redefinition of professional roles was evident, as most service providers have little to no autonomy in administering HCV tests and rely on nurses or other healthcare professionals for this task. Intersectionality considerations were also important for providers, who described extensively how their social identity and lived experience intersects with their professional role. Many mentioned leveraging their lived experience of HCV, drug use or homelessness to relate more authentically with clients and enhance the effectiveness of HCV testing interventions.

The findings highlight several key areas for enhancing service delivery in needle and syringe programs, along with potential implementation strategies to support the uptake of point-of-care HCV testing. Notable strategies include enhancing education and awareness of HCV testing among people who inject drugs, potentially through educational meetings and materials that explain the testing process—including the distinctions between antibody and RNA testing—and that emphasize the benefits of point-of-care tests through stories and testimonials. Offering financial or material incentives to individuals who get tested, receive their results, and are linked to care appears to be critical. Equally essential is fostering inclusive and comfortable environments that cater to the diverse needs of people who inject drugs and providing comprehensive training for providers, including stigma reduction to create a non-judgmental and supportive environment and tailored approaches to engage women and transgender individuals in HCV care. Employing service providers with personal experience of HCV or drug use, with different social identities, and favoring peer-based approaches could enhance the trust and comfort levels of those considering testing. The integration of services is vital, creating pathways that seamlessly incorporate HCV testing with other services offered by the organization and establishing partnerships with other healthcare providers to facilitate referrals and comprehensive care [24, 73, 75].

Incorporating an intersectionality lens into implementation science is an emerging practice [36, 42, 45]. While the field has developed robust methodologies for addressing implementation challenges, there is an increasing consensus on the necessity of equity-focused approaches [35, 38, 41–43, 76, 77]. Our study contributes to this body of evidence by showcasing the application of an intersectionality lens throughout various phases of the implementation research process—from identifying relevant research questions to employing a multilayered approach to data analysis and interpretation. This approach demonstrates how to consider intersecting identities and experiences in the research process. The use of intersectionality should be complemented by community-based participatory research approaches [78] for the co-design of implementation strategies, enabling a deeper understanding of the nuanced realities of marginalized populations and the complex dynamics of real-world settings.

Our study has some limitations. First, the research was conducted within a single, relatively well-resourced urban community organization, which may affect the generalizability of the findings to other needle and syringe programs or harm reduction settings, both within urban areas and more broadly in rural or less-resourced settings. Second, as with any qualitative research, the themes and insights derived are interpretive, and the analysis is influenced by the researchers’ biases and perspectives, which may introduce confirmation bias or overlook other relevant themes. Third, while employing an intersectionality lens provides a comprehensive framework for analyzing the influence of overlapping social identities and systemic structures, it also adds complexity to data interpretation. There is a risk that some intersecting factors, particularly more subtle or less recognized forms of discrimination and privilege, may not have been fully captured or articulated by the participants, potentially leading to an incomplete analysis of how intersectionality influences point-of-care HCV testing. There is a need for future studies conducted with larger sample sizes, to ensure a more complete representation of different identity groups. Furthermore, the approach applied in this study should be refined and not considered a one-size-fits-all solution. It should be tailored to different contexts and populations to better address their unique needs and challenges.

## Conclusions

In this study, applying an intersectionality lens provided detailed insights into the barriers and enablers influencing the uptake of point-of-care HCV testing within needle and syringe programs. The results demonstrate how various forms of stigma associated with HCV, substance use, and overlapping social factors such as gender, ethnicity, and socioeconomic status significantly affect access to care for people who inject drugs. These insights highlight the urgent need for tailored strategies that effectively address stigma, enhance communication skills among providers, and cultivate an inclusive environment that supports equitable access to HCV care. This study contributes to the growing recognition of intersectionality as a valuable tool in equity-focused implementation research.

## Data Availability

Data that support the findings of this study are available upon reasonable request.

## Abbreviations

HCV: Hepatitis C virus
RNA: Ribonucleic acid
STBB: Sexually transmitted and blood-borne infections
TDF: Theoretical Domains Framework
WHO: World Health Organization
